# Re-Assembly of the Genome of *Francisella tularensis* Subsp. *holarctica* OSU18

**DOI:** 10.1371/journal.pone.0003427

**Published:** 2008-10-17

**Authors:** Daniela Puiu, Steven L. Salzberg

**Affiliations:** Center for Bioinformatics and Computational Biology, University of Maryland, College Park, Maryland, United States of America; Indiana University, United States of America

## Abstract

*Francisella tularensis* is a highly infectious human intracellular pathogen that is the causative agent of tularemia. It occurs in several major subtypes, including the live vaccine strain holarctica (type B). *F. tularensis* is classified as category A biodefense agent in part because a relatively small number of organisms can cause severe illness. Three complete genomes of subspecies holarctica have been sequenced and deposited in public archives, of which OSU18 was the first and the only strain for which a scientific publication has appeared [1]. We re-assembled the OSU18 strain using both *de novo* and comparative assembly techniques, and found that the published sequence has two large inversion mis-assemblies. We generated a corrected assembly of the entire genome along with detailed information on the placement of individual reads within the assembly. This assembly will provide a more accurate basis for future comparative studies of this pathogen.

## Introduction

*Francisella tularensis* is highly infectious human pathogen that can cause illness after exposure to as few as 10 organisms [2]. It was examined for military purposes in Japan, the former Soviet Union, and the United States at various times during the mid-20^th^ century [2], and it is still considered a serious bioterrorism threat. It has four recognized subspecies, two of which (subsp. *tularensis* and *holarctica*) cause most human illness, and there is substantial genomic conservation among all types [3].

Because of its importance as a biothreat agent, *F. tularensis* has been the subject of intense genome sequencing efforts: seven strains have been completely sequenced and at least twelve more are in progress. For many of these strains, the original raw sequence data (“reads”) and the completed genome sequences are publicly available; however, in no case is the assembly itself – the complete specification of the placement of the reads in the assembled genome – available. The NCBI Assembly Archive [4] was developed to store information on how a particular assembly was constructed, but it does not yet contain any assemblies for *F. tularensis* species. Therefore, if a question arises about the correctness of the genome sequence, one cannot examine the experimental evidence that underlies the genome to see if there is any ambiguity or uncertainty.

## Methods

As part of a project to develop assays to detect *F. tularensis* [5], we re-assembled the OSU18 strain and compared our assembly to the published sequence. We found two major inversions, both of which we have corrected as described below.

The sequencing of OSU18 generated 68,462 reads using ABI 3730 capillary sequencers, covering the genome at approximately 23× coverage [1]. The whole-genome shotgun reads were originally assembled using the Atlas [6] and Phrap [7] assemblers into 132 contigs. The low quality regions and gaps were closed by a variety of finishing methods [1], and the final genome was a single circular chromosome containing 1,895,727 base pairs (bp) (GenBank accession CP000437). The shotgun reads (but not the finishing reads) are available for download from NCBI Trace Archive, and these were downloaded and used to reconstruct the genome.

We used a variety of assembly strategies, including the Celera Assembler [8] and AMOScmp [9]. We obtained the best result using AMOScmp on a modified version of the reference sequence, CP000437. Prior to assembly, the reads were re-trimmed by the Figaro vector trimming program [10]. This procedure produced just 12 contigs spanning the genome, which were then validated using the nucmer [11] and amosvalidate [12] programs. By design, this assembly was a reconstruction of CP000437, and by placing all the reads along the original genome, we were able to examine the paired-end data for any inconsistencies.

Our final re-assembly of *F. tularensis* OSU18 comprises a single, circular chromosome that is identical to the original genome except for two large inversions, described below. The assembly contains both the consensus sequence and the underlying reads, and is available for download from our site at ftp://ftp.cbcb.umd.edu/pub/data/F_tularensis_holarctica_OSU18. The sequence is available from the GenBank database as accession number TPA: BK006741.

## Results and Discussion

The 68,462 *F. tularensis* OSU18 reads were generated from clone inserts averaging 2000–3000 bp; the paired-end information was provided to our assembly methods. We first assembled the reads *de novo* using the Celera assembler (CA), which generated 163 contigs with an average length of 13 Kbp and a maximum length of 455 Kbp.

The numbers of *de novo* contigs generated by Petrusino et al. (134) and by CA (163) are relatively high for a bacterial genome sequenced at 23× coverage. This fragmentation was caused by the large number of repeats spread throughout this genome. These repeats include insertion sequences that are responsible for genomic rearrangements among different Francisella strains [13].

Two large chromosomal inversions were evident in the alignment of our *de novo* assembly to the CP000437 sequence. The inversions correspond to coordinates 16,336–21,562 (∼5 kbp) and 167,086–184,936 (∼18 kbp) in CP000437. In each case, the regions are flanked by two 950-bp oppositely-oriented repeats. Because these 950-bp repeats are longer than any individual reads, they are not spanned by any single sequence; such repeats are a common cause of mis-assemblies.

**Figure 1:**
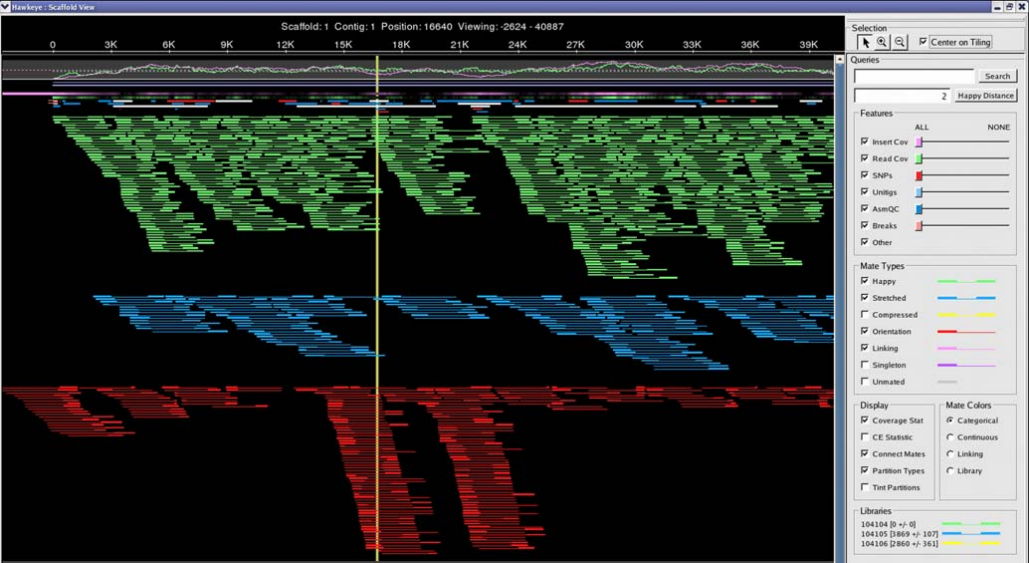
Hawkeye view of the AMOScmp assembly, showing pileup of mis-oriented reads in the chromosome region from position 16,336 to 21,562 (5227 bp). In the figure, pairs of sequences from the same clone insert (“mate pairs”) are shown as line segments, with the rectangle at end of a segment representing each sequence in the pair. Green lines represent “happy” mate pairs; i.e., pairs of reads that are oriented correctly and that are separated by the expected distance. Blue lines show “stretched” mate pairs in which the reads are slightly too far apart. Red lines show mis-oriented mate pairs, in which each read is facing in the wrong direction. The pileup of red lines in the lower central portion of the figure indicates the position of the erroneous inversion. Reads inside the inverted region, as well as their mates outside the inversion, are all mis-oriented.

**Figure 2:**
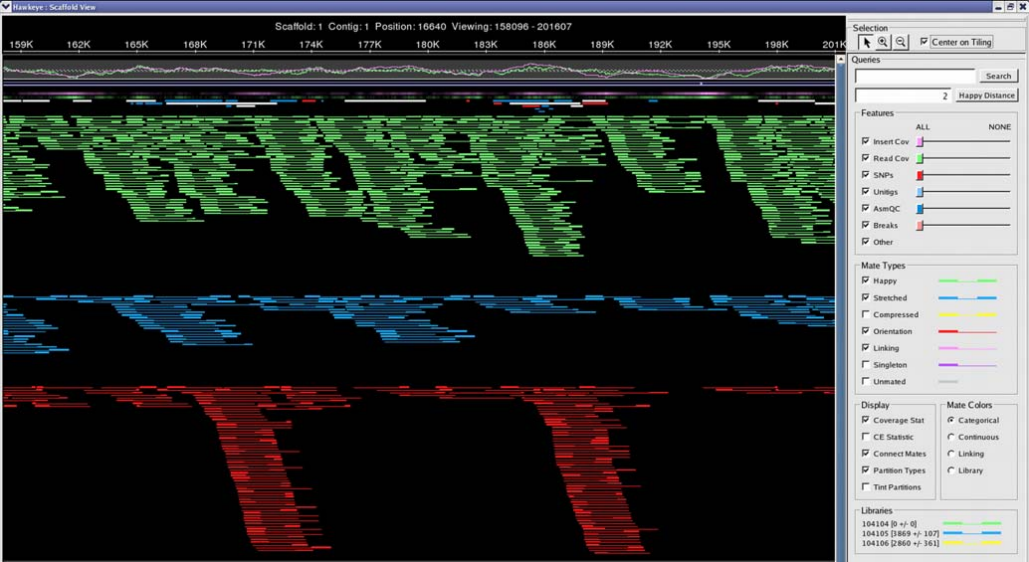
Hawkeye view of the AMOScmp assembly, showing pileup of mis-oriented reads (shown in red) in the chromosome region from position 167,086 to 184,936 (17,851 bp). See Figure 1 legend for detailed description.

We inspected the suspected inversions in the AMOScmp reconstruction of CP000437 using the Hawkeye assembly viewer [14], which highlights regions that might be mis-assembled. As shown in Figures 1 and 2, the assembly based on CP000437 contains large pile-ups of mis-oriented reads (shown in red) flanking each inversion. Each red pair of reads in the figures represents two paired end sequences that are mis-oriented: in paired-end sequencing, both ends of a genomic fragment are sequenced, with the 5′ ends of the pair on the outer ends of the fragment. In the resulting assembly, the two sequences in a pair must be pointing “inward”; e.g., the positions of the reads must be in opposite orientations with the 5′ ends outermost and the 3′ ends innermost.

When a section of a genome is incorrectly inverted, the assembly will contain a pile-up of mis-oriented pairs of reads (mate pairs) at either end of the inversion. This is precisely the situation shown in Figures 1 and 2. For the 5 kbp inversion in Figure 1, we found 42 mis-oriented mate pairs (84 reads); and for the 18 kbp inversion in Figure 2, we found 38 mis-oriented mate pairs (76 reads). (A complete list of these mate pairs with their Trace Archive identifiers is provided as Supplemental Table S1.) These orientation violations can be corrected by reversing the sequence that is flanked by each pile-up.

Once we identified the precise boundaries of the inversions, we corrected them and created a new genome sequence. We then ran AMOScmp using the new sequence, which produced just 7 contigs, with 7 gaps totaling 5,973 bp. (Recall that the finishing reads were not available in the Trace Archive, so some missing data was expected.) When we examined the new assembly in Hawkeye, all four piles of mis-oriented reads were gone and no significant inconsistencies were found in the assembly, confirming our inversion hypothesis.

### Conclusion

Even complete genomes sometimes contain assembly errors [15], and no genome assembly software is perfect. Without detailed information on the placement of individual reads within an assembly, these errors are difficult but not impossible to find. By re-assembling genomes and examining them carefully, we can identify and correct mis-assemblies. Correcting genome assemblies is vital to future work on these organisms, particularly for efforts to design assays such as those used in microbial forensics [16] that require unique markers for each bacterial strain.

## Supporting Information

Table S1 Trace Archive identifiers of paired sequences spanning the boundaries of each inversion in the originally published genome of F. tularensis OSU18 (GenBank accession CP000437).
(0.04 MB DOC)
