# Supplementary material for: Re-Assembly of the Genome of Francisella tularensis Subsp. holarctica OSU18
Source: PLoS One. 2008 Oct 17;3(10):e3427. doi: 10.1371/journal.pone.0003427 (PMC2561293; doi:10.1371/journal.pone.0003427)
Supplement: Table S1 — Trace Archive identifiers of paired sequences spanning the boundaries of each inversion in the originally published genome of F. tularensis OSU18 (GenBank accession CP000437). (0.04 MB DOC) [file pone.0003427.s001.doc]

**Supplemental Table 1: Trace Archive identifiers of paired sequences spanning the boundaries of each inversion in the originally published genome of *F. tularensis* OSU18 (GenBank accession CP000437).** Pairs of reads are shown together, one pair per line. Mate pairs should be in opposite orientations, but all pairs shown here are in the same orientation, due to mis-assemblies in the original sequence. Our corrected assembly is available under GenBank acceccsion BK006741.

**Inversion 1: coordinates 16336-21562 of CP000437**

Forward Reverse

ti number start end dir ti start end dir dist

1348836912 13843 14623 + 1348835261 20644 21470 + 7627

1348840931 13843 14598 + 1348839460 20649 21391 + 7548

1348854592 13843 14623 + 1348853596 20699 21508 + 7665

1348840446 13848 14538 + 1348838968 20698 21444 + 7596

1348809525 13896 14517 + 1348809611 20241 20916 + 7020

1348851026 13933 14849 + 1348850942 20571 21429 + 7496

1348859384 14285 15024 + 1348860807 20448 21200 + 6915

1348848786 14305 15170 + 1348848699 20468 21347 + 7042

1348820432 14325 15132 + 1348820617 19992 20839 + 6514

1348835761 14438 15249 + 1348837388 20658 21436 + 6998

1348878440 14503 15459 + 1348878897 19401 20356 + 5853

1348814762 14554 15355 + 1348814941 20329 21136 + 6582

1348852621 14790 15253 + 1348853357 19449 20031 + 5241

1348876876 14890 15848 + 1348875692 19381 20355 + 5465

1348877077 14984 15700 + 1348877902 20616 21333 + 6349

1348845336 15052 15530 + 1348845213 20385 21081 + 6029

1348818656 15066 15526 + 1348818349 20649 21380 + 6314

1348862994 15145 15411 + 1348862402 20381 21099 + 5954

1348877373 15162 16201 + 1348876273 18377 19423 + 4261

1348828687 15307 15867 + 1348831013 20112 20990 + 5683

1348877787 15363 16322 + 1348876965 19840 20798 + 5435

1348822065 15376 16290 + 1348822147 16814 17724 + 2348

1348842765 15404 16274 + 1348841657 19801 20639 + 5235

1348836072 15415 16063 + 1348834410 19237 19971 + 4556

1348837844 15510 16212 + 1348836273 20212 20975 + 5465

1348807786 16502 17349 - 1348807700 23047 23883 - 7381

1348858016 16602 17440 - 1348859317 22146 22990 - 6388

1348864433 16605 17456 - 1348864519 22170 22994 - 6389

1348816149 16661 17504 - 1348816369 22299 23121 - 6460

1348808984 16661 17427 - 1348808867 23047 23897 - 7236

1348836736 16747 17525 - 1348838373 22212 23053 - 6306

1348876077 16762 17628 - 1348875160 22263 23143 - 6381

1348876043 16771 17629 - 1348875125 25116 26043 - 9272

1348871217 16938 17880 - 1348871793 23751 24686 - 7748

1348828002 17059 17881 - 1348830154 22631 23451 - 6392

1348833135 17273 17974 - 1348834975 21877 22698 - 5425

1348846321 17415 18208 - 1348846250 21577 22435 - 5020

1348863973 17453 18253 - 1348864146 22107 22960 - 5507

1348874063 18180 19089 - 1348873231 23842 24756 - 6576

1348846285 18353 19157 - 1348846196 21715 22570 - 4217

1348878610 18520 19494 - 1348878332 21623 22603 - 4083

1348832599 18929 19327 - 1348835409 21568 22180 - 3251

**Inversion 2: coordinates 167086-184936 of CP000437**

Forward Reverse

ti number start end dir ti start end dir dist

1348874211 163548 164541 + 1348875407 183787 184893 + 21345

1348878218 164118 165098 + 1348878682 182935 183925 + 19807

1348875158 164226 165144 + 1348876096 182389 183263 + 19037

1348877479 164314 165255 + 1348876746 181344 182297 + 17983

1348858978 164695 165554 + 1348857700 183585 184412 + 19717

1348840055 164704 165557 + 1348838491 183608 184449 + 19745

1348839102 164761 165607 + 1348837529 183825 184560 + 19799

1348845457 164938 165711 + 1348847297 183401 184141 + 19203

1348857028 165081 165796 + 1348858112 183474 184196 + 19115

1348808274 165253 165982 + 1348808368 183860 184602 + 19349

1348870288 165311 166277 + 1348870738 182222 183171 + 17860

1348872604 165313 166323 + 1348873343 181949 182962 + 17649

1348829198 165436 166299 + 1348831043 182944 183838 + 18402

1348829912 165577 166390 + 1348827697 183178 183986 + 18409

1348865818 165610 166381 + 1348865511 183274 184040 + 18430

1348821694 165699 166733 + 1348821603 182315 183342 + 17643

1348849466 165755 166450 + 1348849484 183292 183772 + 18017

1348818243 165827 166669 + 1348817791 183901 184664 + 18837

1348821438 165873 166889 + 1348821344 181840 182892 + 17019

1348829285 166021 166860 + 1348827249 183029 183875 + 17854

1348817452 166032 166764 + 1348816885 182595 183167 + 17135

1348830944 166103 166953 + 1348828974 183392 184203 + 18100

1348817983 166171 166864 + 1348819033 183076 183335 + 17164

1348876372 166304 166790 + 1348875469 181785 182829 + 16525

1348807470 166429 166992 + 1348810542 183728 184306 + 17877

1348818016 167206 168171 - 1348818369 185339 186224 - 19018

1348871223 167362 168305 - 1348871784 187158 188086 - 20724

1348807833 167463 168309 - 1348807750 186004 186904 - 19441

1348808710 167523 168115 - 1348808794 186054 186829 - 19306

1348813907 167591 168210 - 1348814029 186443 187162 - 19571

1348862535 167730 168533 - 1348862030 185830 186598 - 18868

1348865773 167876 168624 - 1348865457 185037 185721 - 17845

1348810004 168100 168325 - 1348809935 185525 185962 - 17862

1348855112 168166 168580 - 1348853817 186028 186648 - 18482

1348844385 168764 169258 - 1348847658 185018 185601 - 16837

1348819489 169086 169906 - 1348819653 185453 185778 - 16692

1348877037 169298 170332 - 1348877860 185080 186126 - 16828

1348872398 170077 171051 - 1348872943 185476 186448 - 16371

Key:

ti: NCBI Trace Archive trace identifier (mate pairs listed together on each line)

start: trace alignment to CP000437 start position

end: trace alignment to CP000437 end position

dir: direction of trace alignment to CP000437, either forward (+) or reverse (-) strand

dist: distance between the beginning of the first and the end of the second alignment
